# Moving toward a standardized regional anesthesia approach in clavicle surgery

**DOI:** 10.1016/j.bjane.2025.844711

**Published:** 2025-11-16

**Authors:** Martin Julian Schaefer, Martin Zoremba

**Affiliations:** Academic Teaching Hospital of the University of Marburg, Klinikum Siegen, Department of Anesthesiology and Intensive Care Medicine, Siegen, Germany

*Dear Editor*,

Over the past few years, we have closely followed the ongoing debate regarding the optimal regional anesthesia technique for clavicle surgery. Here, we would like to share our clinical experience and outline the technical details of a combined supraclavicular nerves and C5 root block that we have implemented in our institution.

The routine use of peripheral nerve blocks in this context remains controversial due to the complex innervation of the clavicle, which involves both the cervical and brachial plexuses.^1^, ^2^, ^3^ Typically, surgical treatment of these injuries is performed under general anesthesia with systemic opioid-based analgesia, and establishing a standardized approach for peri- and postoperative pain management in clavicle fractures and acromioclavicular (AC) joint dislocations remains challenging. Considering the proven benefits of regional anesthesia, such as superior analgesia, reduced opioid consumption, and enhanced postoperative recovery, optimizing its application for clavicle injuries is both necessary and clinically valuable.^4^

To address this gap, we conducted a randomized controlled trial (German Clinical Trial Register, DRKS00017286) evaluating the analgesic potential of a combined selective blockade of the supraclavicular nerves and the C5 nerve root compared with single C5 root blockade and systemic analgesia alone. The study protocol and detailed results are available in the German Clinical Trials Register and have been published as a preprint (https://doi.org/10.1101/2025.09.22.25336305). A total of 56 patients were randomized into three groups. The first group received general anesthesia with a combined nerve block of the supraclavicular nerves and the C5 root (n = 19), the second group received general anesthesia only with a C5 root block (n = 18), and the third group had general anesthesia with systemic analgesia (control group, n = 19). Primary outcomes were postoperative pain scores (Numeric Rating Scale, NRS) and opioid consumption within 24 hours. Secondary outcomes included diaphragmatic excursion measured by M-mode ultrasound and oxygen saturation to assess phrenic nerve function. Patients with a combined blockade of the supraclavicular nerves and the C5 nerve root reported no pain during the first postoperative hour, significantly less than the C5-only and the control group. No additional opioid was required, which was also significantly lower compared with the C5-only and the control group. Phrenic nerve palsy was more frequent in the group with a combined blockade of the supraclavicular nerves and the C5 root, although oxygen saturation remained unaffected. By performing a selective blockade technique using a limited volume of local anesthetic, we initially aimed to avoid phrenic nerve involvement. However, these patients showed a significant reduction in diaphragmatic excursion during the first postoperative hour, with an average decline of about 50% compared with baseline values. Despite this decrease in diaphragmatic movement, no oxygenation impairment was observed. Nevertheless, our findings may not apply to patients with pre-existing pulmonary disease or obesity, in whom compensatory capacity may be reduced. Given that clavicle injuries predominantly affect athletic individuals with few comorbidities, this technique appears to be safe in this population. Based on these findings, combined selective regional anesthesia of the supraclavicular nerves and the C5 root may represent an effective approach for reducing postoperative pain and opioid consumption. Therefore, this approach has been adopted routinely for clavicle and AC joint surgeries in our department, where it is currently performed in approximately 100 cases per year.

The nerve block is performed preoperatively in awake patients, who are placed in the supine position with the head of the bed elevated by approximately 15° to 30°. The head is turned about 45° to the contralateral side and slightly elevated from the surface using a small head ring, identical to the standard positioning used for the interscalene brachial plexus block. The block is performed using an ultrasound-guided in-plane technique with a linear probe and a 50 mm needle through a single skin puncture (Fig. 1). The supraclavicular nerves are identified anterior to the prevertebral fascia and middle scalene muscle, embedded in the cervical fascia. For effective blockade, 5 mL of local anesthetic (2.5 mL ropivacaine 0.75% + 2.5 mL prilocaine 1%) is injected to ensure fascial spread. The needle is then advanced under ultrasound guidance to the C5 nerve root in the interscalene gap, where an additional 5 mL of the same anesthetic mixture is administered. In our experience, this technique is straightforward, safe, and can be completed within a few minutes by an anesthesiologist experienced in ultrasound-guided nerve blocks.

**Figure 1:**
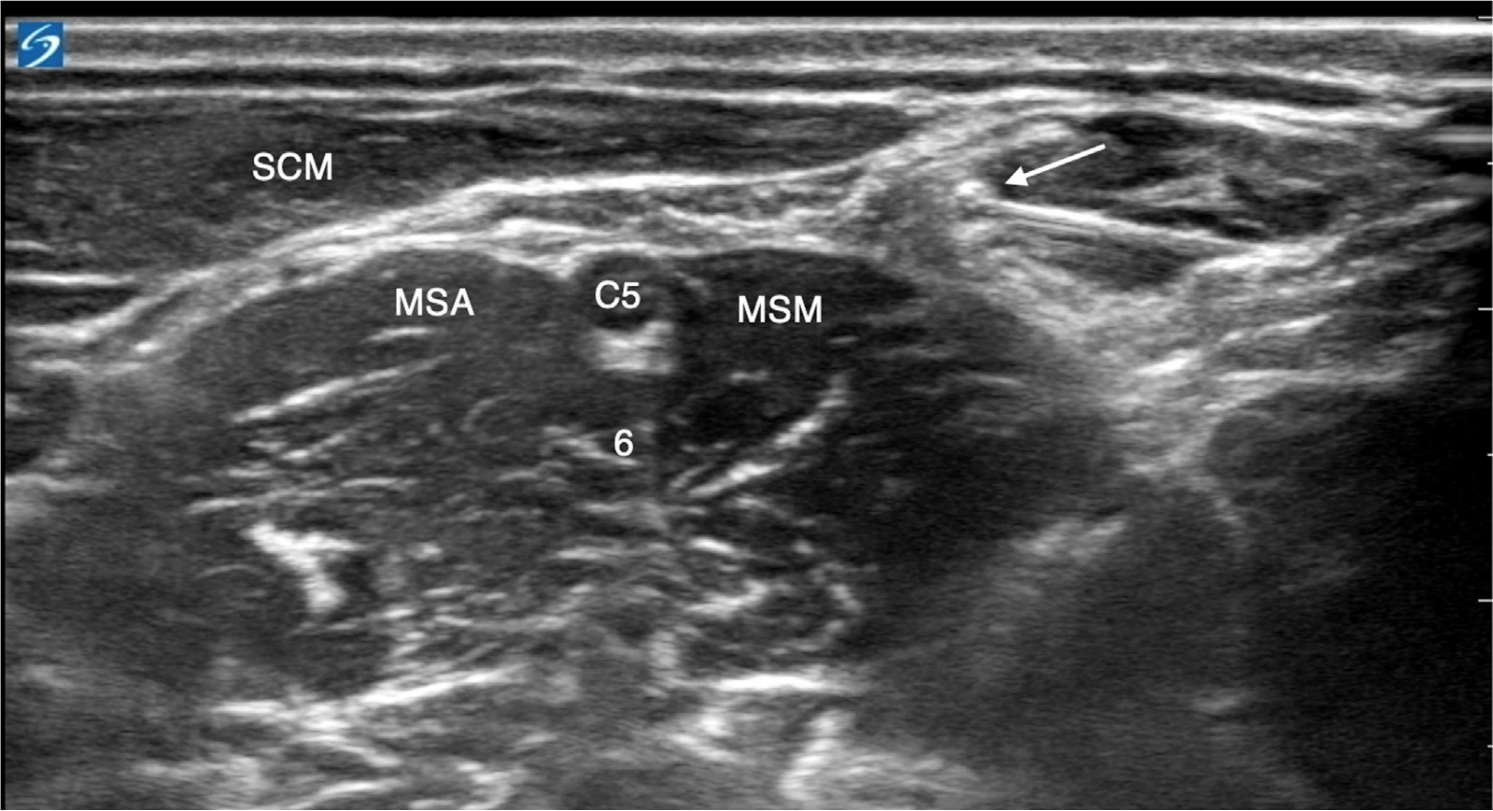
Selective blockade of the supraclavicular nerves and the C5 root. Arrow, Needle positioning at the level of the supraclavicular nerves; C5, C5 nerve root; (6), C6 nerve root; SCM, Sternocleidomastoid Muscle; MSA, Anterior Scalene Muscle; MSM, Middle Scalene Muscle.

We acknowledge the methodological limitations of our study, including its small sample size, open-label design, and short follow-up, which may limit the strength and generalizability of the results. As noted previously, our study did not include patients with significant comorbidities or severe pulmonary diseases. These factors should be taken into careful consideration when interpreting the findings of this blockade technique that may be associated with changes in phrenic nerve function. Nevertheless, the consistent clinical effectiveness and ease of reproducibility of this approach in daily practice are encouraging and suggest that it may serve as a model for wider adoption. Further well-designed randomized trials are warranted to evaluate its efficacy and safety and to support broader standardization. Future research should also explore its potential as a sole anesthetic technique without additional general anesthesia and its possible impact on faster postoperative recovery. With advances in ultrasound-guided regional anesthesia, approaches such as the clavipectoral fascial plane block have also emerged as promising alternatives for postoperative analgesia and merit further investigation.^5^

## Trial registration

German Clinical Trial Registry DRKS ID: DRKS00017286, https://drks.de/search/en/trial/DRKS00017286/entails, date of registration May 20, 2019.

## Ethics committee approval

Ethical Commission Westfalen-Lippe, Gartenstrasse 210‒214, Münster/Germany, protocol number 2018-645-f-S, date of approval: March 14, 2019, chairperson Univ.-Prof. Dr. med. W.E. Berdel.

## Data availability statement

The datasets generated and/or analyzed during the current study are available from the corresponding author upon reasonable request.

## AI assistance disclosure

No AI tools were used in the preparation of this manuscript. The authors take full responsibility for the content.

## Conflicts of interest

The authors declare no conflicts of interest.
